# Osteoprotegerin and tumor necrosis factor-related apoptosis-inducing ligand as prognostic factors in rheumatoid arthritis: results from the ESPOIR cohort

**DOI:** 10.1186/s13075-015-0705-3

**Published:** 2015-07-29

**Authors:** Rachel Audo, Claire Daien, Laura Papon, Cédric Lukas, Olivier Vittecoq, Michael Hahne, Bernard Combe, Jacques Morel

**Affiliations:** Department of Rheumatology, Lapeyronie Hospital, Montpellier University, 371 avenue doyen Giraud, 34295 Montpellier, France; Montpellier University, 163 rue Auguste Broussonnet, 34000 Montpellier, France; Institut de Génétique Moléculaire de Montpellier, CNRS-UMR 5535, 1919 Route de Mende, 34293 Montpellier, France; Department of Rheumatology and CIC/CRB 1404, Rouen University Hospital, Inserm U 905, Institute for Research and Innovation in Biomedicine, 1, rue de Germont, 76031 Rouen, France; Academic Medical Center, University of Amsterdam, Meibergdreef 9, 1105 AZ Amsterdam, The Netherlands

## Abstract

**Introduction:**

We previously reported that low ratio of osteoprotegerin (OPG) to tumor necrosis factor-related apoptosis-inducing ligand (TRAIL) was associated with Disease Activity Score in 28 joints (DAS28) remission at 6 months in patients with early rheumatoid arthritis (RA). Here, we aimed to evaluate the value of baseline OPG/TRAIL ratio in predicting clinical and radiological outcomes in patients with early RA in the ESPOIR cohort.

**Methods:**

OPG and TRAIL serum concentrations were assessed in the ESPOIR cohort patients. Patients with definite RA were included in this study. Patients were excluded if they had high erosion score at baseline (>90^th^ percentile) or received biological therapy during the first 2 years of follow-up. Data were analyzed by univariate analysis and multivariate logistic regression to predict 1-year DAS28 remission and 2-year radiographic disease progression.

**Results:**

On univariate analysis of 399 patients, OPG/TRAIL ratio at baseline was significantly lower in patients with than without remission at 1 year (*p* = 0.015). On multivariate logistic regression including age, gender, body mass index and DAS28, low OPG/TRAIL ratio was independently associated with remission at 1 year (odds ratio 1.68 [95 % confidence interval 1.01–2.79]). On univariate analysis, high OPG/TRAIL ratio at baseline was associated with rapid progression of erosion at 2 years (*p* = 0.041), and on multivariate logistic regression including age, anti-citrullinated protein antibody positivity and C-reactive protein level, OPG/TRAIL ratio independently predicted rapid progression of erosion at 2 years.

**Conclusions:**

OPG/TRAIL ratio at baseline was an independent predictor of 1-year remission and 2-year rapid progression of erosion for patients with early rheumatoid arthritis. Thus, OPG/TRAIL ratio could be included in matrix prediction scores to predict rapid radiographic progression. Further confirmation in an independent cohort is warranted.

## Introduction

Rheumatoid arthritis (RA) is a frequent autoimmune disease, with a prevalence of 0.3 to 1 % worldwide. Numerous therapeutic options include conventional synthetic disease-modifying anti-rheumatic drugs (DMARDs), tumor necrosis factor inhibitors, tocilizumab, abatacept or rituximab. Most patients primarily receive conventional synthetic DMARDs because early intensive therapy is not cost-effective [1]. However, a subgroup of patients is at risk of radiographic disease progression and has a low chance of achieving remission with conventional synthetic DMARDs. These patients usually have high levels of rheumatoid factor (RF) and high titers of anti-citrullinated protein antibodies (ACPA), very high disease activity and/or early radiographic joint damage [2]. Biomarkers beside these usual prognostic factors that could identify patients at risk of radiographic progression and inadequate response to conventional synthetic DMARDs would allow for more intensive therapy andameliorating the disease course in this targeted population.

The cytokine tumor necrosis factor-related apoptosis-inducing ligand (TRAIL) was initially described for its ability to trigger cell death in a somewhat tumor-selective manner. The TRAIL system is probably one of the most complex members of the TNF family because of the large number of receptors to which TRAIL can bind but also because of the signaling pathways engaged. TRAIL can interact with five different receptors: four membrane-anchored receptors, TRAIL-R1 (DR4), -R2 (DR5), -R3 (DcR1) and -R4 (DcR2), and a soluble decoy receptor, osteoprotegerin (OPG). Because of the diversity of TRAIL receptors, multiple proprieties were described. TRAIL can trigger apoptosis as well as proliferation and differentiation depending on the cell type (reviewed in [3]). The first report linking TRAIL with arthritis came from a mouse study with a collagen-induced arthritis model [4, 5]. Studies investigating the role of TRAIL in RA mostly focused on the therapeutic potential of TRAIL, especially RA fibroblast-like synoviocytes (FLSs), because hyperplastic RA FLSs have tumor-like features [6]. However, we found that TRAIL induces apoptosis only in a subset of RA FLSs and induces proliferation in surviving cells [7]. This finding challenged the use of TRAIL for targeting hyperproliferative FLSs, and despite numerous reports describing the effect of TRAIL on RA, its role in pathogenesis is still not fully clarified [3].

OPG is also a decoy receptor for receptor activator of nuclear factor B ligand (RANKL) [8]. OPG has been demonstrated to be involved in bone erosion and bone remodeling [9], and it was recently shown that genetic variant in OPG is associated with progression of joint destruction in RA [10]. RANKL and its receptor RANK play a key role in regulating osteoclastogenesis. Indeed, RANKL stimulates differentiation of osteoclasts via RANK signaling. Competing with RANK for RANKL binding, OPG is able to prevent osteoclastogenesis activity [8, 11]. In addition, OPG inhibits TRAIL-induced apoptosis by binding to TRAIL [11]. Conversely, TRAIL blocks OPG-mediated inhibition of osteoclastogenesis. Thus, OPG and TRAIL may inhibit their respective biological functions.

Because the role of TRAIL in patients with RA was not well established, we performed a prospective pilot study to measure serum levels of OPG and TRAIL in patients with early RA (<2 years) and investigated their association with various clinical parameters [12]. Low OPG/TRAIL ratio at baseline was associated with remission (Disease Activity Score in 28 joints [DAS28] <2.6) at 6 months, which suggests that the ratio of OPG to TRAIL might be a predictive factor for remission in early RA [12]. In the present study, we evaluated the independent value of OPG/TRAIL ratio in predicting DAS28 remission at 1 year according to the European League Against Rheumatism (EULAR) criteria and rapid radiographic disease progression (at 2 years) to identify patients who would benefit from early intensive therapy. Patients were from a large and well-documented cohort of early RA, the Etude et Suivi des POlyarthrites Indifférenciées Récentes (ESPOIR) cohort.

## Methods

### ESPOIR cohort

The ESPOIR cohort is a prospective multicenter observational study of patients 18–70 years old who have early arthritis under the umbrella of the French Society for Rheumatology. The protocol of the ESPOIR cohort study was approved in July 2002 by the ethics committee of Montpellier University (number 020307). All patients gave their signed informed consent to be included in the study.

Patients were recruited if they had inflammatory arthritis in at least two swollen joints lasting for 6 weeks to 6 months, with the potential to develop into RA. The population and methods of the ESPOIR cohort are detailed elsewhere [13]. Treatment by rheumatologists followed the standard of care and patients were followed every 6 months during the first 2 years, then every year. At baseline and each visit, clinical and biological data relevant to the management of early arthritis were recorded. Patients underwent radiography of the hand, wrist (face) and foot (face and oblique) at baseline, then at 1 and 2 years. X-ray films were scored by use of the van der Heijde-modified Sharp score (mSharp score) [14] for radiographic disease progression.

The ESPOIR cohort included 813 patients. In this ancillary study, we included patients fulfilling the American College of Rheumatology-European League Against Rheumatism (ACR-EULAR) 2010 criteria for RA at inclusion. We excluded patients with a history of lymphoma and neoplasia (n = 13) because of a known relation between TRAIL and tumor genesis and those with the highest erosion scores (mSharp erosion score >90^th^ percentile; n = 62). Indeed, radiographic erosion at baseline is a well-characterized factor of further radiographic progression [15], and the EULAR task force recommended prompt use of biological therapy in these rare cases [1]. The 90^th^ percentile of mSharp erosion score corresponded to 4 points. We also excluded patients who received biological therapy in the first 2 years (n = 121) because it strongly affects radiographic disease progression.

### Serum assays

Blood samples were taken for investigation of C-reactive protein (CRP) level and erythrocyte sedimentation rate (ESR). Serum samples were collected at enrollment and immediately stored at -80 °C in a single biologic resource centre. A central laboratory determined levels of anti-citrullinated cyclic peptide (anti-CCP) antibodies (anti-CCP2; DiaSorin, Saluggia [Vercelli], Italy; positive >50 U/ml) and rheumatoid factor (RF) (Ménarini France, Rungis Cedex, France; positive >9 IU/ml) with enzyme-linked immunosorbent assay (ELISA).

### Determination of OPG and TRAIL serum levels

OPG and TRAIL serum levels were measured by using commercially available ELISA kits (human OPG Quantikine ELISA kit, R&D Systems, Minneapolis, MN, USA; and human TRAIL ELISA kit, Diaclone, Besançon, France) and expressed in pg/ml. RF depletion did not alter results for OPG or TRAIL concentrations (data not shown).

### Radiographic disease progression

Rapid radiographic progression was defined by at least a 5 point per year increase in total mSharp score, which corresponds to a 10-point increase at 2 years. This cutoff has been previously used [16, 17]. Because erosion and joint-space narrowing are almost similar to total mSharp score, we defined rapid progression of erosion or rapid progression of joint-space narrowing as at least a 5-point increase in erosion score or joint-space narrowing score at 2 years.

### Statistical analysis

The values of OPG and TRAIL showed a skewed distribution, and normality was obtained after natural logarithmic (log) transformation. OPG to TRAIL ratio (OPG/TRAIL ratio) was used as previously described [12]. Student *t* and chi-square tests were used to compare baseline characteristics for 1) patients with or without DAS28 remission at 1 year and 2) patients with or without rapid radiographic disease progression at 2 years (total mSharp score increase ≥10 or erosion score ≥5 or joint-space narrowing score ≥5). Odds ratios (ORs) were obtained by logistic regression, with 1-year DAS28 remission and 2-year rapid radiographic progression as dependent variables. All covariates associated at the 20 % level (*p* <0.20) on univariate analysis were included in the multivariate logistic regression model as potential confounders and selected by stepwise multiple regression. A receiver-operating characteristic (ROC) curve was plotted to identify a cutoff value of OPG/TRAIL ratio associated with 2-year rapid radiographic progression [18]. True-positive patients were those with high OPG/TRAIL ratio and rapid radiographic progression, and true-negative patients were those with low ratio OPG/TRAIL ratio and without rapid radiographic progression. The best possible OPG/TRAIL ratio threshold was determined by using the highest Youden Index [(specificity + sensitivity) − 1] [19]. Statistical analysis involved use of PASW v18 (SPSS Inc., Chicago, IL, USA).

## Results

### Patient characteristics

The characteristics of all RA patients included in ESPOIR cohort (fulfilling ACR-EULAR 2010 criteria) and the 399 patients included in this study are in Table 1. Briefly, patients included in this study and all ESPOIR cohort patients were similar except for total mSharp score because we excluded patients with the highest erosion score at baseline.

**Table 1:** Baseline characteristics of all patients with rheumatoid arthritis (RA) responding to 2010 ACR-EULAR criteria included in the ESPOIR cohort and RA patients included in this study

Characteristics	All RA patients (n = 641)	Patients in the study (n = 399)
Age (years)	48.1 ± 12.6	48.4 ± 11.9
Female (%)	78	81
Body mass index (kg/m^2^)	25.1 ± 4.7	25.2 ± 4.6
Rheumatoid factor positivity (%)	55	51
Anti-citrullinated protein antibody positivity (%)	50	45
DAS28(ESR)-4v	5.4 ± 1.2	5.3 ± 1.2
HAQ score	1.0 ± 0.7	1.0 ± 0.7
Total mSharp score	5.29 ± 7.5	3.77 ± 4.4
Steroid use (%)	13	14

Data are mean ± SD or percentage

*RA* rheumatoid arthritis, *DAS28(ESR)-4v* Disease Activity Score in 28 joints, calculated with erythrocyte sedimentation rate and four values, *HAQ* Health Assessment Questionnaire, *mSharp score* van der Heijde-modified Sharp score

### Age, gender, DAS28 and OPG/TRAIL ratio are associated with DAS28 remission at 1 year

On univariate analysis, age, gender, DAS28 and OPG/TRAIL ratio at baseline differed between patients with and without DAS28 remission at 1 year (Table 2). Patients in remission were younger, less often female and had lower DAS28 at baseline than patients with active disease at 1 year. Moreover, body mass index and ESR were lower but not significantly for patients in remission than with active disease at 1 year (*p* = 0.06 and *p* = 0.07, respectively) (Table 2). Low OPG/TRAIL ratio (logOPG/TRAIL ratio <25^th^ percentile) was associated with remission at 1 year (OR = 1.76 [95 % confidence interval (95 % CI) 1.11–2.81]) (Table 2). This association was mainly explained by OPG. Indeed, low OPG (Q <25e percentile versus ≥25e percentile) was associated with 1 year remission (OR = 1.76 [1.10–2.81], *p* = 0.017) whereas TRAIL was not. Of note, OPG/TRAIL ratio correlated with DAS28 at baseline (r = 0.10; *p* = 0.04).

**Table 2:** Association of main baseline characteristics of RA patients with DAS28 remission at 1 year (univariate analysis)

	DAS28 remission at 1 year		*P* value	OR (95 % CI)
	yes	no		
Age (years)	46.5 ± 12.6	49.6 ± 11.3	0.01	0.98 (0.96–0.99)
Females (%)	75	86	0.01	0.49 (0.29–0.82)
Body mass index (kg/m^2^)	24.6 ± 4.4	25.5 ± 4.6	0.06	0.96 (0.91–1.00)
Rheumatoid factor positivity (%)	51.3	51.1	0.97	1.01 (0.67–1.51)
Anti-citrullinated protein antibody positivity (%)	46.8	43.7	0.55	1.14 (0.75–1.71)
mSharp score	3.53 ± 4.02	3.75 ± 4.49	0.61	0.99 (0.94–1.04)
DAS28(ESR)-4v	4.98 ± 1.18	5.49 ± 1.16	0.00	0.69 (0.57–0.823)
logCRP (log-mg.l^−1^)	1.01 ± 0.47	1.03 ± 0.49	0.73	0.93 (0.59–1.44)
ESR	25 ± 22	29 ± 24	0.07	0.99 (0.98–1.00)
Steroids (%)	87.8	85.6	0.53	1.21 (0.66–2.22)
Use of cDMARD (%)	78.7	79.6	0.83	1.95 (0.57–1.57)
logOPG/TRAIL ratio Q <25 (%)	31.4	20.6		1.76 (1.11–2.81)^a^*
logOPG/TRAIL ratio Q25–75 (%)	41.7	51.7	0.015	
logOPG/TRAIL ratio Q >75 (%)	26.9	23.7		1.19 (0.74–1.89)^b^

Data are mean ± SD (median) or percentage. Student *t* test for continuous variables and chi-square test for categorical variables

*RA* rheumatoid arthritis, *DAS28* Disease Activity Score in 28 joints, *OR* odds ratio, *95 % CI*, 95 % confidence interval, *cDMARD* conventional disease-modifying anti-rheumatic drug (used at least at two visits), *CRP* C-reactive protein, *DAS28(ESR)-4v* Disease Activity Score in 28 joints, calculated with erythrocyte sedimentation rate (ESR) and four values, *mSharp score* van der Heijde-modified Sharp score, *OPG* osteoprotegerin, *TRAIL* tumor necrosis factor-related apoptosis-inducing ligand, *Q <25* quartile <25^th^ percentile, corresponding to 0.932, *Q >75* quartile >75^th^ percentile, corresponding to 1.039

*p <0.05 *p* value for comparison of ^a^Q <25^th^ versus ≥25^th^ percentile and ^b^Q >75^th^ versus ≤75^th^ percentile

### Low OPG/TRAIL ratio independently predicts DAS28 remission at 1 year

On multivariate logistic regression, female sex was associated with DAS28 remission at 1 year (OR = 0.49 [95 % CI 0.28–0.86]) as was low DAS28 at baseline (OR = 0.71 [0.59–0.86]). ESR was not significantly associated (*p* = 0.922) (Table 3). Low logOPG/TRAIL ratio (<25^th^ percentile) was associated with DAS28 remission at 1 year, independent of other factors (OR = 1.68 [1.01–2.79]) (Table 3). This association was mainly explained by OPG. Indeed, low OPG (Q <25e percentile versus ≥25e percentile) was associated with 1 year remission in multivariate model (OR = 1.67 [1.01–2.77]; *p* = 0.047) whereas TRAIL was not.

**Table 3:** Multivariate logistic regression analysis of factors predicting DAS28 remission at 1 year

Variable	OR (95 % CI)	*P* value
Age (per year increase)	0.98 (0.96–1.00)	0.086
Female (versus male)	0.49 (0.28–0.86)	0.013
Body mass index (per kg.m-^2^)	0.95 (0.90–1.00)	0.057
DAS28 (per unit increase)	0.71 (0.59–0.86)	0.001
logOPG/TRAIL ratio Q <25 vs ≥25 (per category increase)	1.68 (1.01–2.79)	0.045

ESR was excluded from the model due to non-significance (*p* = 0.922)

*DAS28* Disease Activity Score in 28 joints, *OR* odds ratio, *95 % CI*, 95 % confidence interval, *OPG* osteoprotegerin, *TRAIL* tumor necrosis factor-related apoptosis-inducing ligand

Of note, OPG/TRAIL ratio at baseline was also associated with DAS28 remission at year 2, although not significantly, in the univariate (*p* = 0.09) and multivariate model (*p* = 0.082; OR = 1.54 [0.95–2.59]).

### RF, anti-CCP antibodies, CRP level, ESR and OPG/TRAIL ratio are associated with rapid radiographic disease progression at 2 years

On univariate analysis, RF and ACPA positivity as well as high CRP level and ESR at baseline were associated with rapid progression of total mSharp score at 2 years (Δtotal mSharp score ≥10, Δerosion mSharp score ≥5 or Δjoint-space narrowing mSharp score ≥5) (Table 4). Concerning total mSharp score, older age was associated with rapid radiographic progression. High OPG/TRAIL ratio (logOPG/TRAIL >75^th^ percentile) was associated with rapid erosion progression (OR = 2.419 [95 % CI 1.136–5.152]) (Table 4).

**Table 4:** Association of main baseline characteristics of RA patients with rapid radiographic progression at 2 years (univariate analysis)

	ΔM24–M0 total mSharp >10		*P* value	OR (95 % CI)	ΔM24–M0 erosion mSharp > 5		*P* value	OR (95 % CI)	ΔM24–M0 joint-space narrowing mSharp >5		*P* value	OR (95 % CI)
	Yes	No			Yes	No			Yes	No		
Age	51.5 ± 10.7	48.2 ± 11.8	0.059	1.03 (0.99–1.05)	51.8 ± 8.9	48.4 ± 11.8	0.108	1.03 (0.99–1.06)	49.4 ± 11.4	48.4 ± 11.7	0.471	1.01 (0.98–1.03)
Female (%)	84.6	81.1	0.543	0.78 (0.35–1.74)	87.5	81.0	0.367	1.64 (0.56–4.83)	83.5	80.9	0.571	1.19 (0.65–2.20)
BMI	25.9 ± 4.9	25.3 ± 4.6	0.245	1.04 (0.98–1.10)	25.1 ± 4.9	25.3 ± 4.6	0.866	0.99 (0.927–1.08)	25.6 ± 4.7	25.2 ± 4.6	0.447	1.02 (0.97–1.07)
RF (%)	69.2	48.2	**0.005**	2.42 (1.29–4.53)	78.1	41.7	**0.005**	3.14 (1.37–7.18)	64.9	46.3	**0.002**	2.15 (1.33–3.47)
ACPA (%)	65.4	41.5	**0.001**	2.67 (1.45–4.92)	75.0	48.9	**0.005**	5.0 (2.106–11.872)	59.8	39.6	**0.001**	2.271 (1.418–3.635)
Total mSharp score	4.0 ± 4.8	3.7 ± 4.4	0.644	1.10 (0.95–1.08)	4.5 ± 5.0	3.7 ± 4.4	0.341	1.04 (0.96–1.12)	3.8 ± 4.8	3.7 ± 4.3	0.837	1.01 (0.95–1.06)
DAS28(ESR)-4v	5.5 ± 1.4	5.3 ± 1.1	0.211	1.2 (0.9–1.5)	5.5 ± 1.5	5.3 ± 1.2	0.331	1.16 (0.86–1.59)	5.3 ± 1.3	5.3 ± 1.1	0.784	1.03 (0.84–1.25)
logCRP level	1.25 ± 0.51	0.98 ± 0.46	**0.000**	3.3 (1.8–6.3)	1.25 ± 0.49	1.00 ± 0.47	**0.004**	1.01 (1.00–1.02)	1.16 ± 0.47	0.96 ± 0.46	**0.001**	2.414(1.445–4.03)
ESR	35 ± 26	28 ± 24	**0.009**	1.01 (1.00–1.02)	36 ± 29	28 ± 24	**0.016**	1.01 (1.00–1.02)	32 ± 25	26 ± 21	**0.030**	1.01 (1.00–1.02)
Steroid use (%)	19.2	13.4	0.264	0.651 (0.305–1.390)	8.0	13.4	0.413	1.66 (0.49–5.65)	83.5	86.6	0.455	0.785 (0.416–1.483)
cDMARD use (%)	89.6	82.2	0.210	1.86 (0.71–4.91)	85.2	83	0.774	1.18 (0.39–3.53)	90.2	80.8	0.041	2.19 (1.03–4.64)
logOPG/TRAIL ratio Q <25 (%)	23.5	25.6		0.894 (0.447–1.787)^a^	12.9	26.4		0.412 (0.140–1.210)^a^	26.0	25.1		1.051 (0.619–1.785)^a^
LogGPG/TRAIL ratio Q25–75 (%)	47.1	50.6	0.685		45.2	50.6	**0.041**		53.1	49.1	0.614	
logOPG/TRAIL ratio Q >75 (%)	29.4	23.8		1.335 (0.695–2.568)^b^	41.9	23.0		2.419 (1.136–5.152)^b^*	20.8	25.8		0.757 (0.432–1.325)^b^

Data are mean ± SD (median) or percentage. Significant values are in bold

*RA* rheumatoid arthritis, *M* month, *mSharp score* van der Heijde-modified Sharp score, *OR* odds ratio, *95 % CI*, 95 % confidence interval, *BMI* body mass index, *RF* rheumatoid factor, *ACPA* anti-citrullinated protein antibodies, *DAS28(ESR)-4v* Disease Activity Score in 28 joints, calculated with erythrocyte sedimentation rate (ESR) and four values, *CRP* C-reactive protein, *cDMARD* conventional disease-modifying anti-rheumatic drug (used at least at two visits),, *OPG* osteoprotegerin, *TRAIL* tumor necrosis factor-related apoptosis-inducing ligand, *Q <25* quartile <25^th^ percentile, *Q >75* quartile >75^th^ percentile

*p <0.05 *p* value for comparison of ^a^Q <25^th^ versus Q ≥25^th^ percentile and ^b^Q >75^th^ versus Q ≤75^th^ percentile

This association was mainly explained by TRAIL with low TRAIL (Q <25^th^ percentile versus Q ≥25^th^) associated with rapid erosion progression (OR = 2.76 [1.30–5.84]; *p* = 0.008) whereas OPG was not associated with radiographic progression.

### OPG/TRAIL ratio is an independent predictor of rapid erosion progression (change in erosion mSharp score >5 at 2 years)

**Fig. 1:**
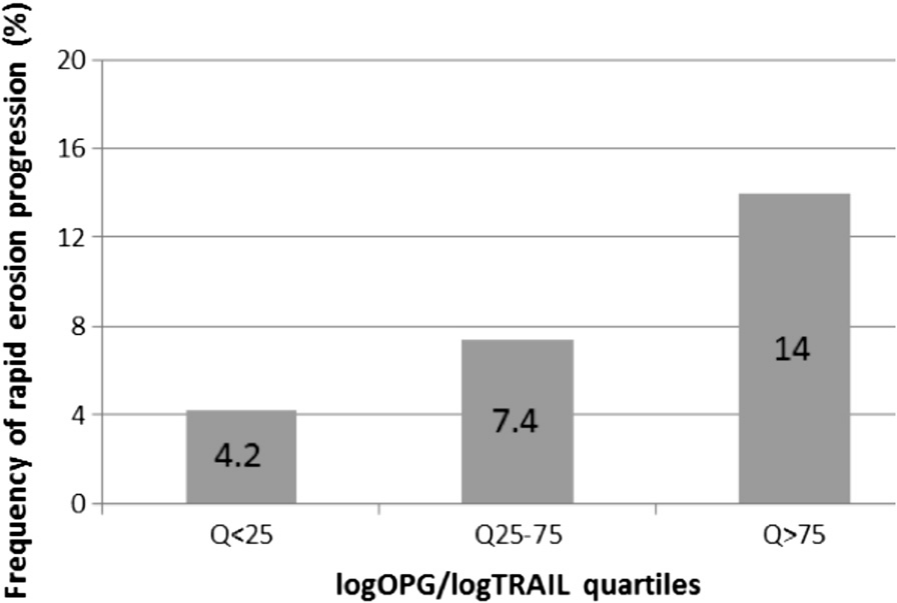
Frequency of rapid progression of erosion for each quartile increase of OPG/TRAIL ratio. Frequency of rapid progression of erosion (change in erosion mSharp score >5 at 2 years) in patients with rheumatoid arthritis at 2 years by baseline logarithmic ratio of quartiles of osteoprotegerin to tumor necrosis factor-related apoptosis-inducing ligand (logOPG/TRAIL) is shown

On multivariate analysis, RF, ESR and total Sharp score at baseline were not significantly associated with rapid erosion progression (*p* = 0.55, *p* = 0.21, *p* = 0.77, respectively) and were excluded from the analysis. Of note, total mSharp score was strongly associated with radiographic progression in all ESPOIR RA patients (*p* <0.001). Thus, excluding patients with the highest erosion scores at baseline, as we did in this study, removed any of the total mSharp score predictive value for rapid radiographic progression. ACPA positivity was highly associated with risk of rapid erosion progression (OR = 3.95 [95 % CI 1.26–12.4]) (Table 5). LogOPG/TRAIL ratio was independently associated with risk of rapid erosion progression (OR = 1.90 [1.03–3.52]) for each quartile increase (Table 5 and Fig. 1). This association was mainly explained by TRAIL with low TRAIL (Q <25^th^ percentile versus Q ≥25^th^) associated with rapid erosion progression (OR = 2.57 [1.14–5.79]; *p* = 0.022) whereas OPG was not associated with radiographic progression in multivariate analysis.

**Table 5:** Multivariate logistic regression analysis of factors predicting rapid erosion progression (ΔM24–M0 erosion mSharp score >5)

Variable	OR (95 % CI)	*P* value
Age (per year increase)	1.06 (0.99–1.07)	0.076
ACPA positivity (versus negativity	3.95 (1.26–12.44)	**0.019**
logCRP (per log-unit)	2.01 (0.83–4.87)	0.121
logOPG/TRAIL ratio (per category increase)	1.90 (1.03–3.52)	**0.041**

*M* month, *mSharp score* van der Heijde-modified Sharp score, *OR* odds ratio, *95 % CI* 95 % confidence interval, *ACPA* anti-citrullinated protein antibodies, *OPG* osteoprotegerin, *TRAIL* tumor necrosis factor-related apoptosis-inducing ligand

Significant values are in bold

Of note, on multivariate logistic regression, age, ACPA positivity and CRP level but not ESR, RF positivity and logOPG/TRAIL ratio were associated with rapid radiographic progression (total mSharp score).

**Fig. 2:**
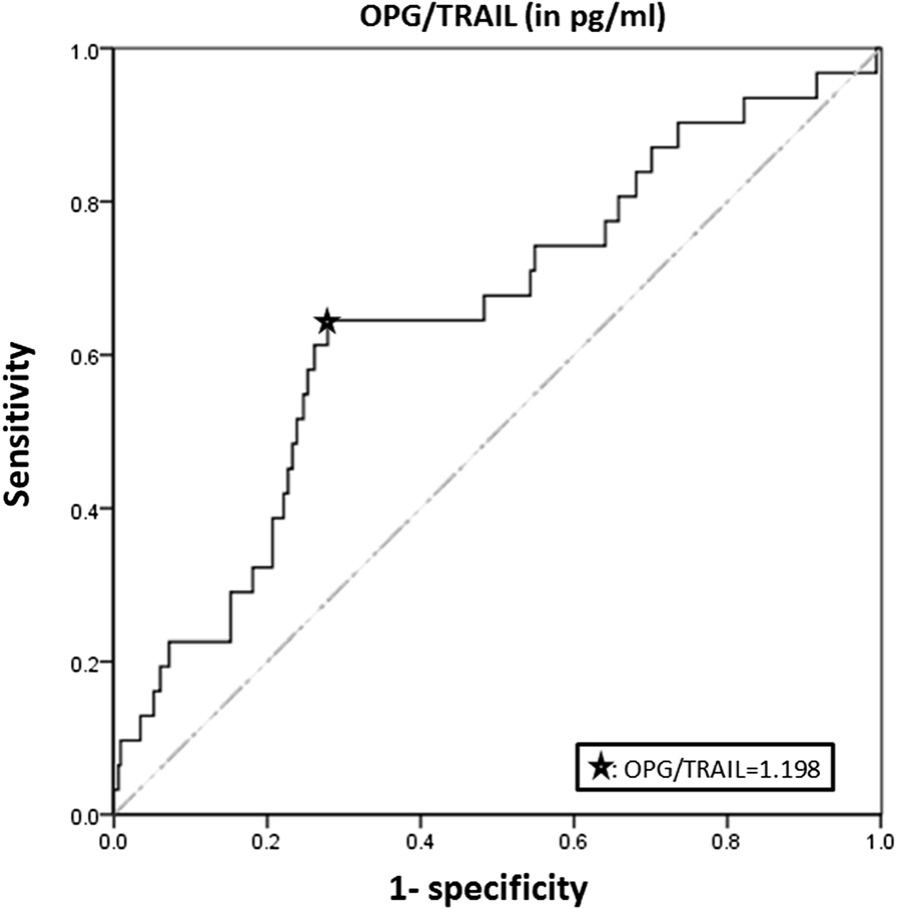
Receiver operating characteristic (ROC) curve to determine the best threshold for OPG/TRAIL ratio to predict rapid erosions. Patients treated with biological therapy during the first 2 years and with radiographic evidence of damage at baseline were excluded. Osteoprotegerin to tumor necrosis factor-related apoptosis-inducing ligand (OPG/TRAIL) ratio (both values expressed in pg/ml). The star shows the 1.198 OPG/TRAIL ratio

Using ROC analysis, we determined that OPG/TRAIL ratio ≥1.198 was the best threshold to predict rapid radiographic progression (Fig. 2), with sensitivity 65 % and specificity 73 % and area under the ROC curve 0.65 ± 0.05 (*p* = 0.005).

## Discussion

We confirmed in a large cohort of early rheumatoid arthritis that low OPG/TRAIL ratio is associated with DAS28 remission at 1 year. In addition, high OPG/TRAIL ratio could predict rapid progression of erosion at 2 years independent of factors known to be associated with radiographic disease progression.

In this study, OPG level at baseline was correlated with disease activity and determined the association between OPG/TRAIL ratio and 1 year remission. OPG has proinflammatory effects that could be mediated by activation of the nuclear factor kappa B (NF-kB) pathway [20, 21], which suggests that OPG plays a role in RA inflammation. Because we found high OPG/TRAIL ratio associated with risk of rapid erosion progression mainly due to low TRAIL values, our results favor a protective effect of TRAIL on bone erosion in RA. This finding could be surprising because TRAIL neutralizes the OPG-inhibitory effect on osteoclastogenesis. However, TRAIL can also have a direct effect on osteoblasts and osteoclasts [3]. In vitro evidence suggests that TRAIL acts on bone remodeling. Indeed, TRAIL-induced apoptosis of osteoblasts differentiated from peripheral blood mononuclear cells in vitro and blocked the differentiation of osteoclasts [22–24]. Thus, in the context of RA, TRAIL could inhibit osteoclast differentiation, thus protecting against erosion.

In contrast, a few studies have investigated the effect of TRAIL on human chondrocytes in vitro [25], which suggests that TRAIL has a marginal impact on cartilage structure. These in vitro observations could suggest that TRAIL is more specifically associated with erosion than joint-space narrowing.

Identifying patients at high risk of radiographic disease progression is useful in daily practice for proposing more intensive therapy. OPG/TRAIL ratio can predict rapid progression of erosion but not rapid radiographic progression with total mSharp score. However, radiographic erosions are the most important parameter predicting long-term total mSharp score, better than joint-space narrowing [26].

The strength of this study is the large number of patients and its prospective design. One limitation is that patients received treatment according to routine care and not a standardized protocol. The evaluation of 2-year radiographic progression was performed under different therapeutics. Therefore, we cannot rule out the possibility of treatment confounders. To partially control for this confounding factor, we excluded patients who received biological therapy during this period. In addition, the relatively low sensitivity and specificity of OPG/TRAIL ratio in predicting rapid progression of erosion, leaves the possibility that our findings could be false positive. A replication in an independent cohort will be warranted. To predict the risk of rapid disease progression, some groups proposed matrix prediction scores [15, 27]. However, these tools need to be constructed with the utmost accuracy to predict rapid radiographic progression. Thus, OPG/TRAIL ratio may be an interesting parameter for inclusion in a matrix.

## Conclusions

In conclusion, we found high OPG/TRAIL ratio associated with lack of disease remission in RA and with rapid progression of erosion. These results support a protective role of TRAIL on bone, probably mediated by its effects on osteoclast differentiation and apoptosis. Measuring serum OPG and TRAIL level to calculate the OPG/TRAIL ratio could help predict patients at high risk of radiographic disease progression.

## Abbreviations

ACPA: anti-citrullinated peptide antibodies
ACR: American College of Rheumatology
CI: confidence interval
CRP: C-reactive protein
DAS28: Disease Activity Score in 28 joints
DMARDs: disease-modifying anti-rheumatic drugs
ELISA: enzyme-linked immunosorbent assay
ESPOIR: Etude et Suivi des POlyarthrites Indifférenciées Récentes
EULAR: European League Against Rheumatism
ESR: erythrocyte sedimentation rate
FLSs: fibroblast-like synoviocytes
HAQ: Health Assessment Questionnaire
mSharp score: van der Heijde-modified Sharp score
OPG: osteoprotegerin
OR: odds ratio
RA: rheumatoid arthritis
RANKL: receptor activator of nuclear factor B ligand
RF: rheumatoid factor
ROC: receiver operating characteristic
TRAIL: tumor necrosis factor-related apoptosis-inducing factor
